# Role of scratches and mat hygiene in wrestling-associated skin infections

**DOI:** 10.1016/j.jdin.2025.11.030

**Published:** 2025-12-29

**Authors:** Talia Thomas, Luke Moore, Angela Moore

**Affiliations:** a Texas Christian University School of Medicine, Fort Worth, Texas; b Arlington Center for Dermatology, Arlington, Texas; c Arlington Research Center, Arlington, Texas; d Baylor University Medical Center, Dallas, Texas

**Keywords:** colonization, general dermatology, infection, medical dermatology, MRSA, nasal carriage, *Staphylococcus*, wrestlers

*To the Editor:* Wrestlers and coaches often attribute increased skin infections to mat cleaning frequency and methods.^1^ However, despite improved facility maintenance, skin and soft tissue infections (SSTIs) continue to plague wrestlers. Since nasal colonization has been implicated in methicillin-resistant *Staphylococcus aureus* (MRSA) infections in surgical site infections,^2^ with the American Academy of Pediatrics citing skin contact and skin trauma as risk factors for SSTI,^1^ impact of skin abrasions on SSTI incidence in wrestlers needs to be explored.

To investigate the impact of scratching frequency and mat cleaning methods on SSTI, a survey was conducted among 109 wrestlers, aged 7-30, from The University of Oklahoma Wrestling Program (OU) and Orange County Regional Training Center (OCRTC). We chose to include both collegiate and club athletes to capture a broader representation of the wrestling community for increased generalizability of our findings; both groups share similar training environments, physical exposures, and SSTI risk factors. The survey was adapted from previously validated questionnaires on athlete health and reviewed for content validity by dermatologists. Recruitment for survey participation was promoted in-person by coaches and targeted social media. The survey included questions about SSTI historyand treatments, scratching frequency and number, skin cultures, known infected contacts, and mat cleaning protocols.

Out of the 33 wrestlers on OUs collegiate team and 120 wrestlers on OCRTC’s club team, we achieved a 71% response rate. Multivariable regression showed that frequent scratches had the highest odds ratio for reported skin infections (OR: 2.70, 95% CI: 0.87-8.37, *P* = .08), indicating a trend toward increased risk (Table I). While not statistically significant, increased scratch frequency demonstrated the highest odds ratio and trend toward significance, with error bars representing 95% Wilson Cis (Fig 1, Supplementary Figs 1 and 2, available via Mendeley at https://data.mendeley.com/datasets/nbcyydj8j5/1). These findings underscore the need for larger studies to confirm these associations, Scratching frequency may be a stronger predictor of SSTI than mat cleaning practices.

**Table I:** Odds ratios and 95% confidence intervals from ordinal logistic regression examining associations between scratch frequency, mat cleaning practices, and reported skin infections among wrestlers

Predictor	Odds ratio	95% confidence interval	*P*-value
Scratched frequently (≥1×/wk)	2.70	0.87-8.37	.08∗
Scratched occasionally	1.57	0.61-4.05	.35
Mats cleaned occasionally	1.40	0.34-5.79	.64
Mats cleaned after every practice	1.22	0.38-3.90	.73
Mats cleaned with ultraviolet C	1.15	0.38-3.49	.80
Mats cleaned using other methods	1.14	0.42-3.09	.80

∗ Trend toward statistical significance.

**Fig 1:**
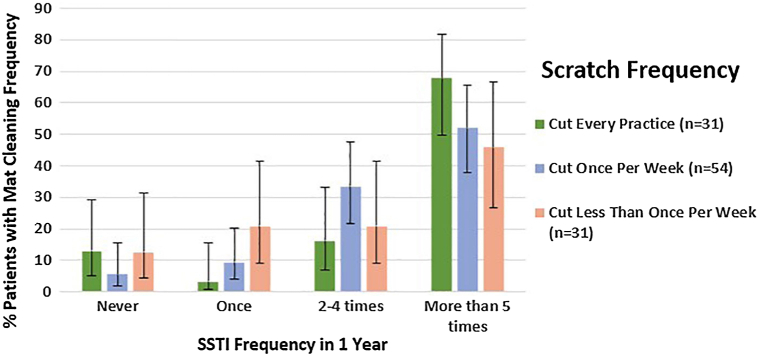
Scratch frequency correlated with SSTI frequency in 1 year.

Since previous studies have identified higher rates of MRSA in those with nasal colonization^2^ or untrimmed fingernails,^3^ and wrestlers commonly receive or give scratches via untrimmed fingernails, reducing MRSA nasal colonization and fingernail carriage among wrestlers might reduce SSTIs. Our conclusions are limited by reliance on self-reported data, which introduces recall bias and potential misclassification of infections and cleaning practices. Additionally, respondents may not have been fully aware of mat cleaning methods or the infection status of their opponents, so findings should be interpreted within the context of observational trends rather than causal relationships. Another limitation included the small sample size, which reduces statistical power.

Our 71% response rate suggests that the data is representative of these specific wrestling populations. However, for broader generalizability and statistical significance, larger surveys across more diverse wrestling communities would be beneficial. Since decolonization in nursing homes^4^ in intensive care units^5^ have decreased SSTIs, intervention with decolonization and chlorhexidine gluconate bathing is speculated to decrease SSTIs in wrestlers. Further prospective and interventional studies are also needed to identify the optimal regimen in wrestlers to prevent MRSA transmission and decrease SSTIs.

## Conflicts of interest

None disclosed.
